# Angiotensin-converting enzyme defines matrikine-regulated inflammation and fibrosis

**DOI:** 10.1172/jci.insight.91923

**Published:** 2017-11-16

**Authors:** Philip J. O’Reilly, Qiang Ding, Samia Akthar, Guoqiang Cai, Kristopher R. Genschmer, Dhiren F. Patel, Patricia L. Jackson, Liliana Viera, Mojtaba Roda, Morgan L. Locy, Ellen A. Bernstein, Clare M. Lloyd, Kenneth E. Bernstein, Robert J. Snelgrove, J. Edwin Blalock

**Affiliations:** 1 Department of Medicine, Division of Pulmonary, Allergy and Critical Care Medicine, University of Alabama at Birmingham, Birmingham, Alabama, USA.; 2 Inflammation Repair and Development, National Heart and Lung Institute, Imperial College London, London, United Kingdom.; 3 Birmingham V.A. Medical Center, Birmingham, Alabama, USA.; 4 Division of Pharmacology, Utrecht Institute for Pharmaceutical Science, Faculty of Science, Utrecht University, Utrecht, Netherlands.; 5 Department of Biomedical Sciences and Department of Pathology, Cedars-Sinai Medical Centre, Los Angeles, California, USA.

**Keywords:** Pulmonology, Fibrosis

## Abstract

The neutrophil chemoattractant proline-glycine-proline (PGP) is generated from collagen by matrix metalloproteinase-8/9 (MMP-8/9) and prolyl endopeptidase (PE), and it is concomitantly degraded by extracellular leukotriene A_4_ hydrolase (LTA_4_H) to limit neutrophilia. Components of cigarette smoke can acetylate PGP, yielding a species (AcPGP) that is resistant to LTA_4_H-mediated degradation and can, thus, support a sustained neutrophilia. In this study, we sought to elucidate if an antiinflammatory system existed to degrade AcPGP that is analogous to the PGP-LTA_4_H axis. We demonstrate that AcPGP is degraded through a previously unidentified action of the enzyme angiotensin-converting enzyme (ACE). Pulmonary ACE is elevated during episodes of acute inflammation, as a consequence of enhanced vascular permeability, to ensure the efficient degradation of AcPGP. Conversely, we suggest that this pathway is aberrant in chronic obstructive pulmonary disease (COPD) enabling the accumulation of AcPGP. Consequently, we identify a potentially novel protective role for AcPGP in limiting pulmonary fibrosis and suggest the pathogenic function attributed to ACE in idiopathic pulmonary fibrosis (IPF) to be a consequence of overzealous AcPGP degradation. Thus, AcPGP seemingly has very divergent roles: it is pathogenic in its capacity to drive neutrophilic inflammation and matrix degradation in the context of COPD, but it is protective in its capacity to limit fibrosis in IPF.

## Abstract 2

ACE degrades the collagen-derived matrikine, acetylate proline–glycine–proline, to limit pulmonary inflammation and promote repair.

## Introduction

Chronic obstructive pulmonary disease (COPD) and idiopathic pulmonary fibrosis (IPF) are 2 severe pulmonary disorders characterized by quite distinct clinical and pathological features, manifesting as alveolar emphysema and bronchiolar inflammation in COPD, versus interstitial fibrosis and honeycombing in IPF (1–3). The pathogenesis of COPD and IPF remain incompletely understood. COPD is associated with a characteristic pattern of chronic inflammation with increased numbers of neutrophils in the airway lumen and increased numbers of macrophages and T and B lymphocytes, though it remains to be clearly defined how this inflammation relates to clinical outcomes and disease progression (4). While inflammation had classically been anticipated to precede fibrosis in IPF, the failure of immunosuppressive therapies has led to a shift in thinking, whereby the disease is now considered to arise in genetically susceptible individuals as a consequence of aberrant wound healing following repetitive alveolar injury (5). Nonetheless, it is anticipated that there are a number of similarities in the etiologies and pathogenesis of the 2 disorders, whereby an accelerated pulmonary senescence coupled with the long-term exposure to noxious chemical agents culminates in chronic, progressive diseases characterized by a compromised regenerative capacity (3, 6–8). The divergences in tissue renewal processes between the 2 diseases ultimately then give rise to the distinct pathologies observed. Effective treatments are lacking for both COPD and IPF, in part due to our still-limited understanding of the mechanisms defining disease progression. Accordingly, a variety of underlying pathogenic mechanisms are being explored, including oxidative stress, protease/antiprotease imbalance, and aberrant repair and remodeling processes (9–11).

A protease/antiprotease imbalance is a hallmark of many chronic lung diseases, with proteases targeting components of the extracellular matrix (ECM) for degradation, disrupting tissue architecture and releasing ECM-derived bioactive signals, termed matrikines, that can perpetuate inflammation (12, 13). The tripeptide proline-glycine-proline (PGP) is one such matrikine, being a neutrophil chemoattractant derived from ECM collagen that exerts its activity by mimicking key sequences found in ELR^+^ chemokines and binding to CXCR1/2 (14). PGP is generated from collagen via the sequential enzymatic activity of matrix metalloproteinases (MMP; with key roles established for MMP-1, -8, and -9) and prolyl endopeptidase (PE) (15). Since neutrophils are a source of the proteases that generate PGP, it is anticipated that this pathway can drive a self-sustaining cycle of neutrophilic inflammation (16). PGP can subsequently be chemically acetylated on its N-terminus (AcPGP), which functions to enhance its chemotactic potential (17–19). Significant quantities of PGP and AcPGP are found in patients with chronic neutrophilic lung diseases such as COPD, cystic fibrosis (CF), and bronchiolitis obliterans syndrome (BOS), peaking with exacerbations of disease and, in some instances, inversely correlating with lung function (14, 15, 20–24).

Recently, we identified an antiinflammatory pathway whereby PGP is degraded during episodes of acute neutrophilic inflammation by the extracellular activity of the enzyme leukotriene A4 hydrolase (LTA_4_H) (17) and showed this degradation to be critical to the timely resolution of inflammation (25). LTA_4_H classically functions intracellularly to catalyze the generation of the proinflammatory lipid mediator leukotriene B_4_ (LTB_4_) (26, 27). LTB_4_ can drive the recruitment and activation of an array of cells, including neutrophils, and is implicated in protection against invading microorganisms, but also in the pathology of multiple diseases (28). Therefore, LTA_4_H represents a highly unusual enzyme with directly opposing pro- and antiinflammatory activities that dictate the amplitude and persistence of neutrophilic inflammation (29). We have subsequently demonstrated that this LTA_4_H-mediated PGP degradation is perturbed by cigarette smoke, contributing to the accumulation of PGP in COPD (17, 21).

While PGP is readily broken down via LTA_4_H, AcPGP is completely protected from degradation (17), and thus, the acetylation process is seemingly a key event in driving the pathogenesis of aforementioned disease states. Accordingly, there has been a sharper focus on processes that define the bioavailability of the acetylated form of PGP. Acetylation of peptides is classically the function of N-acetyl transferases; however, the small size and N-terminal proline of PGP seemingly preclude it from being a favorable substrate for such enzymes (30, 31). Subsequently, however, we have demonstrated that PGP can be chemically acetylated to AcPGP through the action of reactive aldehydes, such as acrolein and acetaldehyde, present in cigarette smoke (18). Reactive aldehydes can also be generated physiologically during episodes of severe inflammation, with acrolein, for example, being a product of lipid peroxidation and the myeloperoxidase- catalyzed (MPO-catalyzed) oxidation of threonine (32, 33). While the processes of PGP acetylation are increasingly being elucidated, it is still unknown as to whether there is an antiinflammatory system in place to degrade AcPGP that is analogous to the PGP-LTA_4_H axis. We previously demonstrated that AcPGP was completely protected from degradation by LTA_4_H but that bronchoalveolar lavage fluid (BALF) from influenza-infected mice possessed some capacity to degrade AcPGP (17). Although this AcPGP-degrading activity was significantly inferior to the capacity of the BALF to degrade PGP (17), the likely disproportionate generation of PGP versus AcPGP and the superior chemotactic activity of the acetylated peptide may seeming make this AcPGP-degrading activity physiologically relevant.

In this study, we looked to characterize and identify the source of this AcPGP-degrading activity and demonstrated that the enzyme responsible was angiotensin-converting enzyme (ACE). Moreover, we reveal how ACE functions during inflammation, in conjunction with LTA_4_H, to ensure efficient resolution of the PGP/AcPGP pathway. Furthermore, we demonstrate that this ACE pathway may be differentially aberrant in COPD versus IPF in a manner that will define the divergent pathogenesis of these disease states.

## Results

### A potentially novel role for the enzyme ACE in degrading AcPGP.

**Figure 1:**
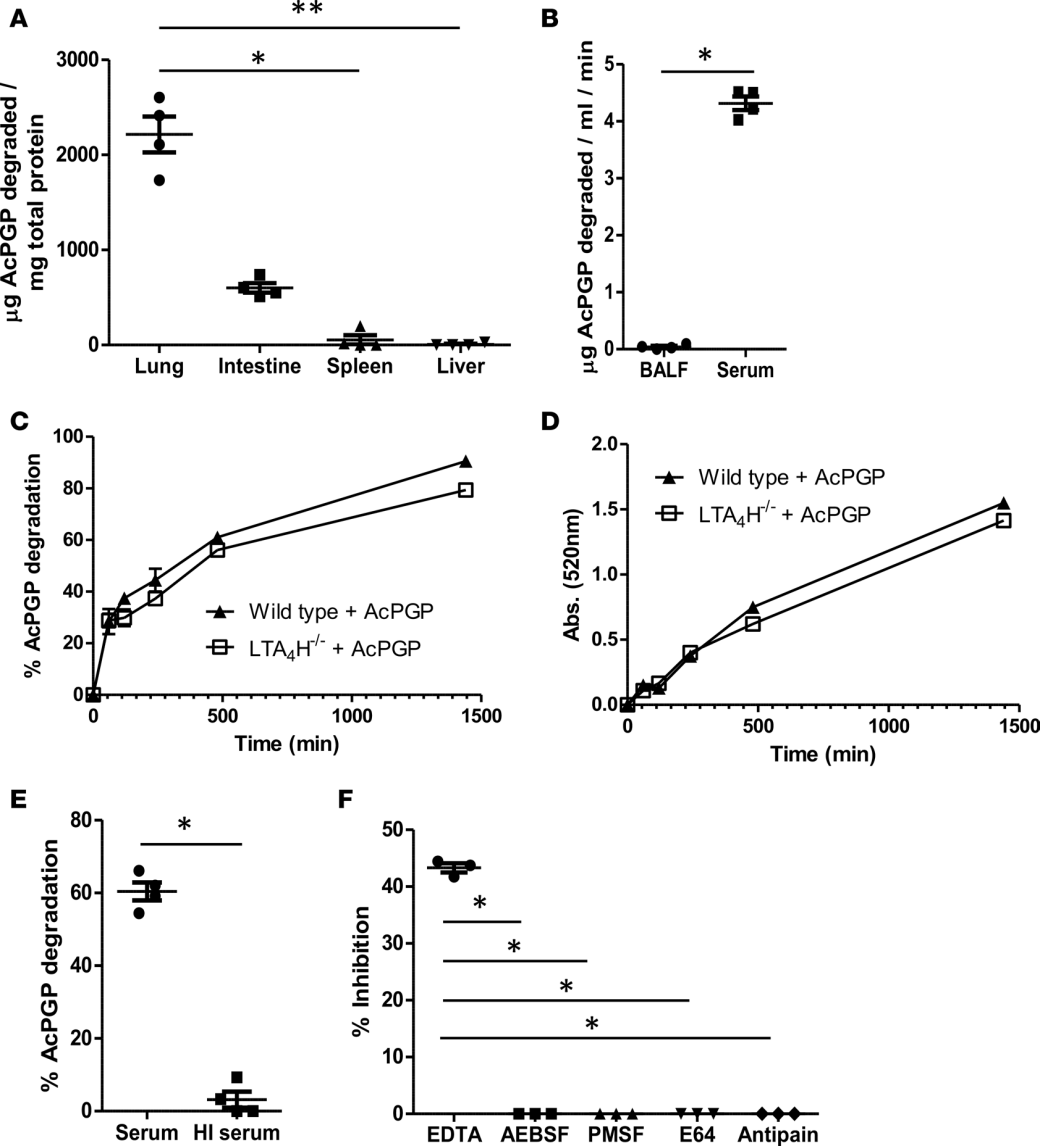
An extracellular enzymatic activity that degrades AcPGP. Tissue homogenates (**A**) or BALF and serum (**B**) from naive Balb/c mice were incubated with AcPGP, and degradation was assessed after 24 hours by mass spectrometry. (**C** and **D**) Serum from naive *lta4h*^–/–^ mice and littermate controls was incubated with AcPGP and degradation assessed at multiple time points by mass spectrometry (**C**) or release of free proline (**D**). Serum from naive Balb/c mice was heat inactivated (HI) for 10 min at 100°C (**E**) or preincubated with selective inhibitors (all at 1 μM) for 30 min at 37°C (**F**) prior to incubation with AcPGP. Degradation of AcPGP was subsequently assessed by mass spectrometry (**E** and **F**). **A** and **B** are from 4 mice/group and representative of 3 experiments. **C**–**F** are triplicates and representative of 2 independent studies. Results depicted as mean ± SEM. *P < 0.05 or **P < 0.01 using Mann–Whitney statistical test (**A**, **B**, and **E**) or Kruskal-Wallis with Dunn’s post test (**F**).

Having previously ascertained that the BALF of influenza-infected mice possessed a modest capacity to degrade AcPGP, we looked to determine the levels and tissue distribution of this potentially novel activity in naive animals. An AcPGP-degrading activity was particularly prevalent in collagen-rich tissues of the lung and intestine (Figure 1A). In order for such an enzymatic activity to be functionally relevant, however, it must be present in an extracellular environment to gain access to AcPGP, and thus, activity of BALF and serum of naive mice was also determined. While weak AcPGP-degrading activity was detectable in naive BALF, it was substantially greater within serum (Figure 1B); therefore, it was deemed that serum was an appropriate matrix from which to further characterize and identify this activity. The capacity of naive serum to degrade AcPGP over time was further interrogated by measuring loss of the peptide by mass spectrometry (Figure 1C) and the corresponding release of free proline via reaction with ninhydrin (Figure 1D). Serum demonstrated a robust capacity to cumulatively degrade AcPGP, with loss of peptide correlating with release of free proline. Importantly, serum from LTA_4_H-KO mice displayed a comparable capacity to degrade AcPGP to that of WT animals (Figure 1, C and D), validating that this AcPGP-degrading activity was distinct from the LTA_4_H-mediated PGP-degrading activity. Heat-inactivating serum prior to incubation with AcPGP largely ablated its capacity to degrade the peptide, supportive of this being an enzymatic activity (Figure 1E). Subsequently, broad-acting inhibitors were assessed for their capacity to inhibit serum-mediated AcPGP degradation to further define the nature of the enzyme responsible (Figure 1F). While classical serine and cysteine protease inhibitors had no effect on the AcPGP-degrading activity of serum, the activity was significantly reduced by EDTA, suggestive that the enzyme responsible was likely a metalloproteinase.

**Figure 2:**
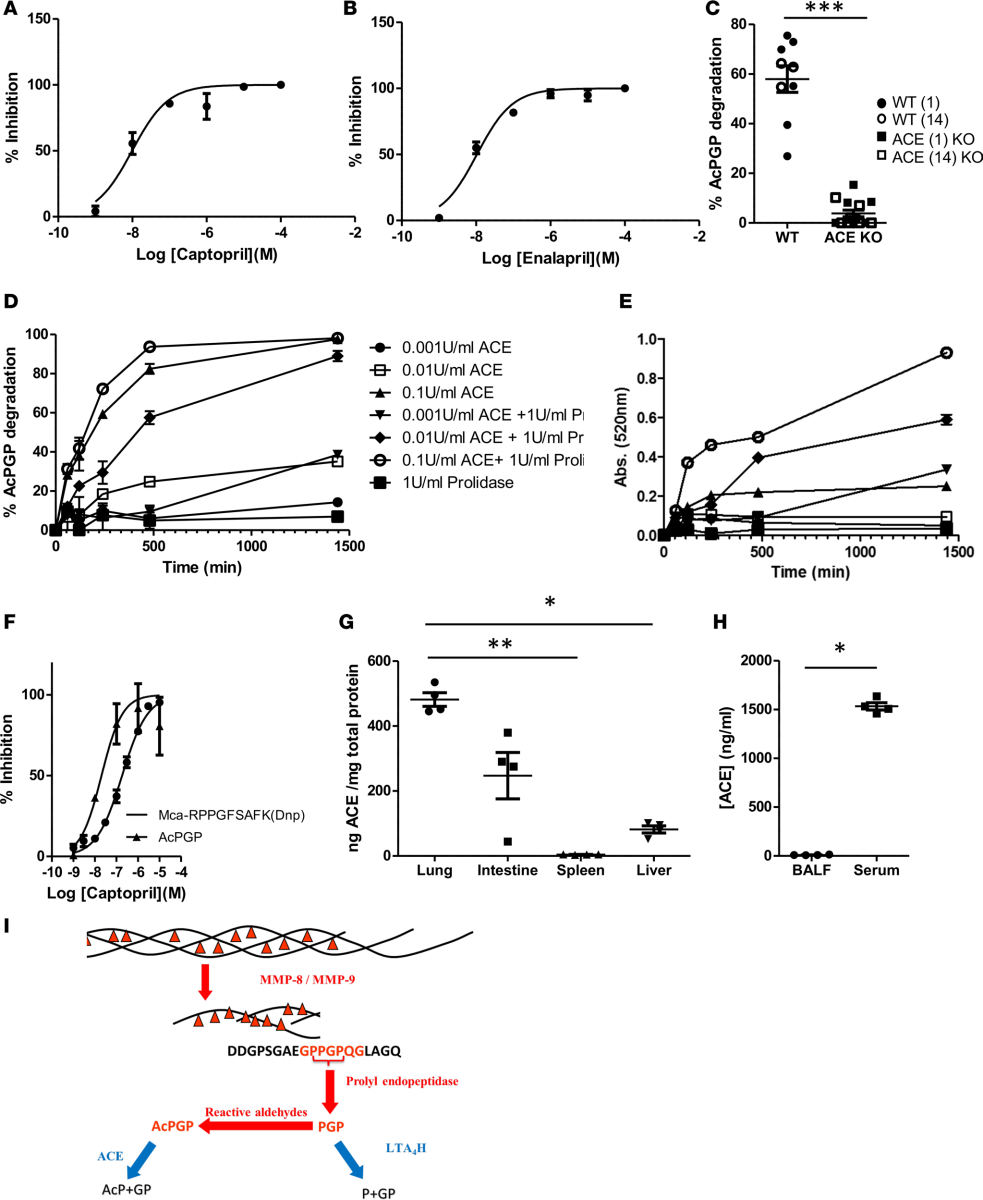
Angiotensin-converting enzyme (ACE) degrades AcPGP. Serum from naive Balb/c mice was preincubated with multiple concentrations (ranging from 1 nM to 100 μM) of captopril (**A**) or enalapril (**B**) for 30 min at 37°C prior to incubation with AcPGP and degradation of peptide was assessed after 24 hours by mass spectrometry. (**C**) Plasma from ACE-KO mice (ACE 1 = null for ACE; ACE 14/14 = null for somatic ACE; *n* = 14) and littermate controls (*n* = 9) were incubated at 37°C with AcPGP and degradation of peptide assessed after 24 hours by mass spectrometry. Recombinant ACE (0.001–0.1 U/ml) and prolidase (1 U/ml) were incubated with AcPGP and degradation assessed at multiple time points by mass spectrometry. ***P < 0.001 (**D**) or release of free proline (**E**). (**F**) Recombinant human ACE was incubated with AcPGP or Mca-RPPGFSAFK(Dnp)-OH and various concentrations of captopril. Degradation of AcPGP was assessed after 24 hours by mass spectrometry and of Mca- RPPGFSAFK(Dnp)-OH fluorometrically. Levels of ACE protein in tissue homogenates (**G**) or BALF and serum (**H**) from naive Balb/c mice were determined by ELISA. (**I**) Schematic of the proteolytic cascade that defines the availability of collagen-derived matrikines PGP and AcPGP. **A**, **B**, **D**, **E**, and **F** are triplicates and representative of 2 independent studies. **G** and **H** are from 4 mice/group and representative of 2 experiments. Results depicted as mean ± SEM. *P < 0.05 or **P < 0.01 using Mann–Whitney statistical test (**C** and **H**) or Kruskal-Wallis with Dunn’s post test (**G**).

While there was clearly an AcPGP-degrading metalloprotease activity that was abundant in the blood, the nature of the enzyme remained unknown, with a limited number of proteases seemingly capable of degrading such a small acetylated peptide. Angiotensin-1–converting enzyme (ACE, EC 3.4.15.1) is a zinc peptidase that is a central component of the renin-angiotensin-aldosterone system (RAAS), wherein it converts angiotensin I into the vasoactive peptide hormone angiotensin II (AngII) (34, 35). However, ACE has also been demonstrated to be capable of degrading a variety of other peptides, including the acetylated tetrapeptide N-acetyl-Ser-Asp-Lys-Pro (AcSDKP; readily metabolizing circulating AcSDKP into inactive AcSD and KP; ref. 36). It was thus rationalized that ACE may be the enzyme that was degrading AcPGP, with this notion supported by the abundance of activity in serum and its dependence on metal ions. To test this hypothesis, the capacity of ACE-specific inhibitors, captopril and enalapril, to abrogate serum AcPGP–degrading activity was assessed. Both captopril (Figure 2A) and enalapril (Figure 2B) displayed a potent and absolute capacity to inhibit serum AcPGP–degrading activity, strongly suggesting that ACE was indeed the enzyme responsible. Importantly, plasma from ACE-KO mice displayed minimal capacity to degrade AcPGP relative to that of littermate controls (Figure 2C), highlighting that degradation of the peptide was almost entirely attributable to ACE. ACE is a dipeptidyl carboxypeptidase, and thus, we rationalized that the enzyme should cleave the GP from AcPGP. However, incubation of serum with AcPGP resulted in the release of free proline (Figure 1D), and this was abrogated if AcPGP was substituted for AcPGG (data not shown), suggestive that a proline is being cleaved from the C-terminus of AcPGP. We therefore hypothesized that an enzymatic cascade was present in serum, whereby AcPGP was initially cleaved by ACE to give rise to AcP and GP, with the GP subsequently being cleaved by prolidase into its individual amino acid constituents. Subsequently, the capacity of recombinant ACE, with and without recombinant prolidase, to degrade AcPGP was assessed by measuring loss of the peptide itself by mass spectrometry (Figure 2D) and generation of free proline via its reaction with ninhydrin (Figure 2E). ACE alone was able to potently and dose-dependently degrade AcPGP (Figure 2D); however, a concomitant release of free proline also required the presence of prolidase (Figure 2E), thus validating the hypothesized proteolytic cascade. We examined captopril inhibition of ACE-mediated AcPGP degradation and found that the 50% inhibitory concentration (IC_50_) was lower than that seen for degradation of an AngI-like ACE substrate (Figure 2F). Levels of ACE in a variety of tissues (Figure 2G) and BALF/serum (Figure 2H) of naive mice were assessed and shown to correlate with AcPGP-degrading activity (Figure 1, A and B). Thus, it appeared that ACE represented a potentially novel antiinflammatory arm of a proteolytic cascade that defined the bioavailability of PGP/AcPGP (Figure 2I).

### Elevated airway ACE levels during episodes of acute pulmonary inflammation facilitate AcPGP degradation.

**Figure 3:**
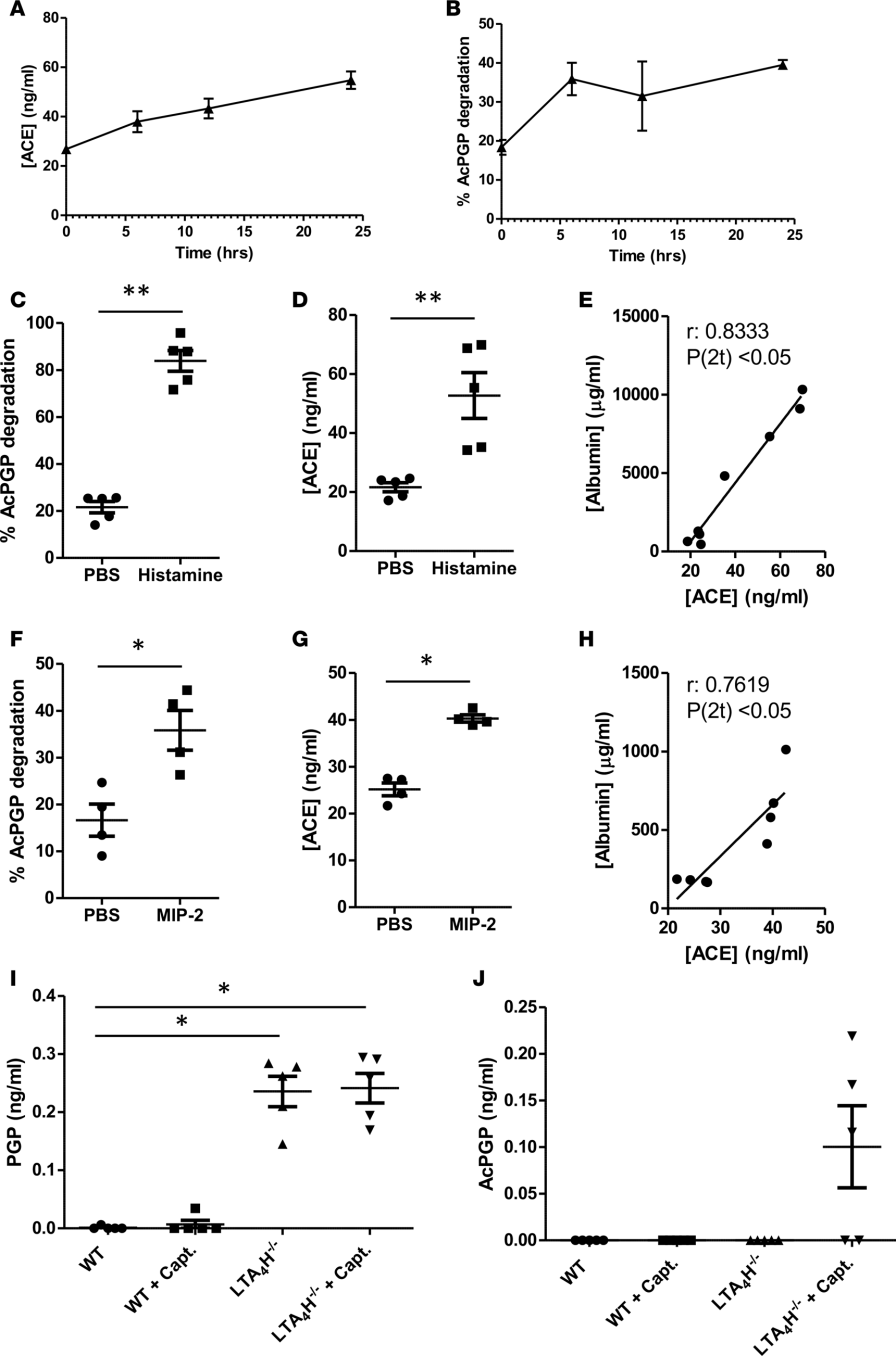
ACE is elevated in BALF following pulmonary challenge to promote AcPGP degradation and is inhibited by captopril. (**A** and **B**) Balb/c mice were administered 10 μg LPS i.n. and culled after 6, 12, or 24 hours. BALF ACE protein levels were determined by ELISA (**A**). BALF from these mice was incubated with AcPGP and degradation assessed after 24 hours by mass spectrometry (**B**). (**C–E**) Naive Balb/c mice were administered histamine i.v. and culled after 30 min. BALF AcPGP–degrading activity was assessed by mass spectrometry (**C**) and ACE protein levels by ELISA (**D**). (**E**) Correlation between BALF ACE protein levels and BALF albumin levels (determined by ELISA). (**F–H**) Balb/c mice were administered 1 μg MIP-2 i.n. and culled after 6 hours. BALF AcPGP–degrading activity was assessed by mass spectrometry (**F**) and ACE protein levels by ELISA (**G**). (**H**) Correlation between BALF ACE protein levels and BALF albumin levels (determined by ELISA). (**I** and **J**) *Lta4h*^–/–^ mice and littermate controls were administered 10 μg LPS i.n. and 2 mg captopril/PBS control i.p. Mice were culled after 24 hours, and levels of PGP (**I**) and AcPGP (**J**) in BALF were assessed by mass spectrometry. Experiments are from 4 (**F** and **G**) or 5 (**A–D**, **I** and **J**) mice/group and representative of 2 experiments. Spearman correlation (**E** and **H**) is representative of 2 experiments, each with at least 4 mice per group. Results depicted as mean ± SEM. *P < 0.05 or **P < 0.01 using Mann–Whitney statistical test (**C** and **H**) or Kruskal-Wallis with Dunn’s post test (**G**). Spearman correlation from 2 experiments with at least 5 mice per group.

We have previously demonstrated that neutrophils are able to generate PGP via the release of proteolytic enzymes that target collagen (16, 17, 25, 37). However, during episodes of acute inflammation, there is the concomitant release of LTA_4_H to ensure that PGP is readily degraded and inflammation efficiently resolved (17, 25). Since liberated PGP is free to be acetylated, which could in turn exacerbate inflammation, we hypothesized that there may also be a synchronized augmentation in BALF ACE during episodes of acute pulmonary inflammation so as to degrade AcPGP generated. Mice were subsequently challenged with intranasal (i.n.) LPS, and at 0, 6, 12 and 24 hours after challenge, BALF AcPGP–degrading activity (Figure 3B) and the concentration of ACE protein (Figure 3A) were assessed. Both BALF AcPGP–degrading activity and levels of ACE itself exhibited a commensurate increase following LPS challenge (Figure 3, A and B). While ACE can be expressed by numerous cell types, the relative abundance of this enzyme in serum led us to speculate that the increase in BALF levels with inflammation may reflect an escalation in vascular permeability and ensuing edema. Early changes in vascular permeability are induced via the direct action of vasoactive agents, such as histamine, on endothelial cells, whereas later changes in vascular permeability are often imparted by the action of neutrophils upon endothelium (38, 39). Subsequently, histamine was administered to mice and, after 30 minutes, resulted in an increased airway edema and a corresponding increase in BALF AcPGP–degrading activity (Figure 3C) and levels of ACE (Figure 3D), which correlated with levels of BALF serum albumin (Figure 3E). Similarly, i.n. administration of neutrophil chemoattractant MIP-2 to mice resulted in augmented airway neutrophilia and an increase in BALF AcPGP–degrading activity (Figure 3F) and levels of ACE (Figure 3G), which again correlated with levels of BALF serum albumin (Figure 3H). Together, these results would suggest that changes in vascular permeability during periods of inflammation may enable ACE to accumulate in the lung to facilitate AcPGP degradation. We subsequently questioned whether abrogation of ACE activity during episodes of acute pulmonary inflammation would enable AcPGP to accumulate in vivo. WT mice were therefore administered i.n. LPS with and without ACE inhibitor captopril, and BALF levels of PGP (Figure 3I) and AcPGP (Figure 3J) were assessed at 24 hours after challenge. Neither PGP nor AcPGP was, however, detectable following LPS exposure, with or without captopril treatment. Previously, we have demonstrated that PGP is efficiently broken down by extracellular LTA_4_H during acute airway inflammation driven by bacterial infection (17), rationalizing the absence of PGP in LPS-treated animals. It was deemed feasible that, following LPS challenge, PGP was being generated but then rapidly degraded by LTA_4_H, providing an insufficient window for AcPGP generation. LTA_4_H-KO mice were therefore also exposed to LPS, with and without captopril treatment, and BALF PGP and AcPGP were measured (Figure 3, I and J). Accordingly, LPS-exposed LTA_4_H-KO mice displayed significant levels of PGP in their airways at 24 hours after challenge (Figure 3I), and the majority of LTA_4_H-KO mice that were coadministered captopril also presented with substantial quantities of AcPGP (Figure 3J). Thus, it would seem that PGP substrate must be allowed to persist for acetylation and generation of AcPGP to occur, which is pertinent given that we have previously demonstrated that cigarette smoke can both abrogate LTA_4_H peptidase activity and acetylate PGP (17, 18); however, ACE displays an important role in degrading any AcPGP that is generated.

### A protective role for AcPGP in fibrosis.

**Figure 4:**
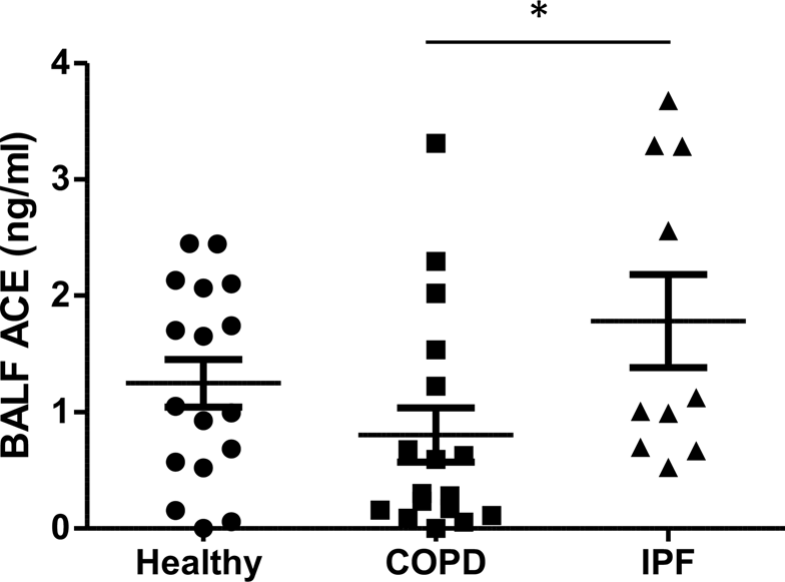
ACE is elevated in BALF of IPF patients. BALF ACE levels in human subjects with IPF (*n* = 10) or COPD (*n* = 17) and healthy controls (*n* = 17) were determined by ELISA. Results depicted as mean ± SEM. *P < 0.05 using unpaired *t* test.

Given the role for ACE described within this study in regulating the AcPGP pathway, it is rational to question the significance of this matrikine in the pathogenesis of IPF. ACE expression has been demonstrated to be elevated in the lungs of IPF patients (40–42), where it is thought to contribute to the development of fibrosis through effects of AngII on myofibroblasts and TGF-β signaling or through degradation of the antifibrotic peptide AcSDKP (43). In keeping with previous studies, our own cohort of IPF patients exhibited elevated BALF levels of ACE relative to COPD patients (Figure 4 and Table 1). Accordingly, PGP and AcPGP were both completely undetectable in the BALF of any of these IPF patients when assessed by mass spectrometry (data not shown). We have previously demonstrated that smokers and COPD patients present with substantial quantities of PGP/AcPGP (21–24) that supports their persistent airway inflammation and pathology, and intriguingly, BALF ACE levels in these patients tended to be lower than that observed in control individuals, though this failed to reach statistical significance (Figure 4).

**Figure 5:**
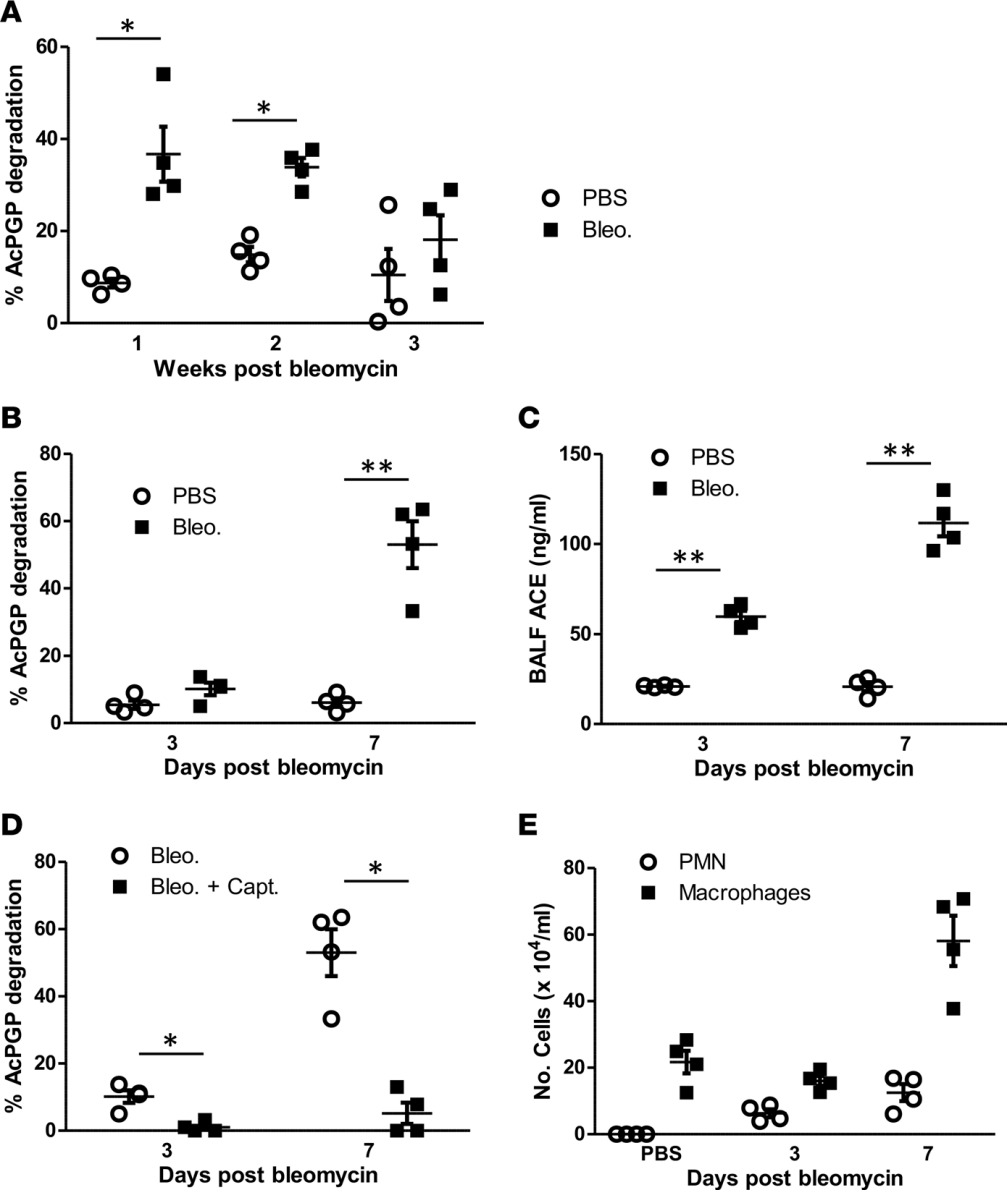
ACE is elevated in BALF following bleomycin exposure to promote AcPGP degradation. (A) C57BL/6 mice were administered bleomycin i.t. and culled after 1, 2, and 3 weeks. BALF was incubated with AcPGP and degradation assessed after 24 hours by mass spectrometry. (**B–E**) C57BL/6 mice were administered bleomycin i.t. and culled after 3 and 7 days. BALF AcPGP–degrading activity was assessed by mass spectrometry (**B**) and ACE protein levels by ELISA (**C**). BALF AcPGP–degrading activity was assessed with and without preincubation with 1 μM captopril (**D**). (**E**) Macrophages and PMNs were enumerated in BALF 3 and 7 days after bleomycin exposure. Experiments are from 4 mice/group. Results depicted as mean ± SEM. *P < 0.05 and **P < 0.01 using Mann–Whitney statistical test.

To further explore the ACE-AcPGP axis in the pathogenesis of IPF, we utilized the bleomycin mouse model that displays some of the features of human disease. In the bleomycin mouse model, there is an initial phase of acute pulmonary inflammation, followed after several days by fibroproliferation and collagen deposition (44, 45). We sacrificed mice 1, 2, and 3 weeks after intratracheal (i.t.) bleomycin exposure and assayed BALF for PGP and AcPGP. In keeping with the IPF patients, bleomycin-exposed mouse BALF contained no PGP or AcPGP at any time point (data not shown). The AcPGP-degrading capacity of bleomycin-exposed mice was subsequently assessed at each time point. BALF AcPGP degrading capacity was significantly greater than control mice 1 and 2 weeks after bleomycin exposure, before declining modestly by week 3, suggesting that this peptidase activity is maximal during acute repair (Figure 5A). In this early inflammatory phase of the bleomycin model, BALF AcPGP–degrading activity (Figure 5B) and ACE protein levels (Figure 5C) reassuringly demonstrated a comparable augmentation. To confirm that ACE was the enzyme degrading AcPGP in the bleomycin model, we demonstrated that addition of captopril to BALF from bleomycin-exposed mice completely abolished its capacity to degrade AcPGP (Figure 5D). The augmented BALF ACE levels observed in bleomycin-exposed mice may reflect an enhanced influx from the vascular compartment, as seen in an acute inflammatory model, but both macrophages and myofibroblasts have been reported as a potential source of this enzyme in fibrosis (46). Accordingly, macrophages were markedly augmented in BALF 1 week after bleomycin exposure (Figure 5E), in keeping with the peak ACE levels at this time point.

**Figure 6:**
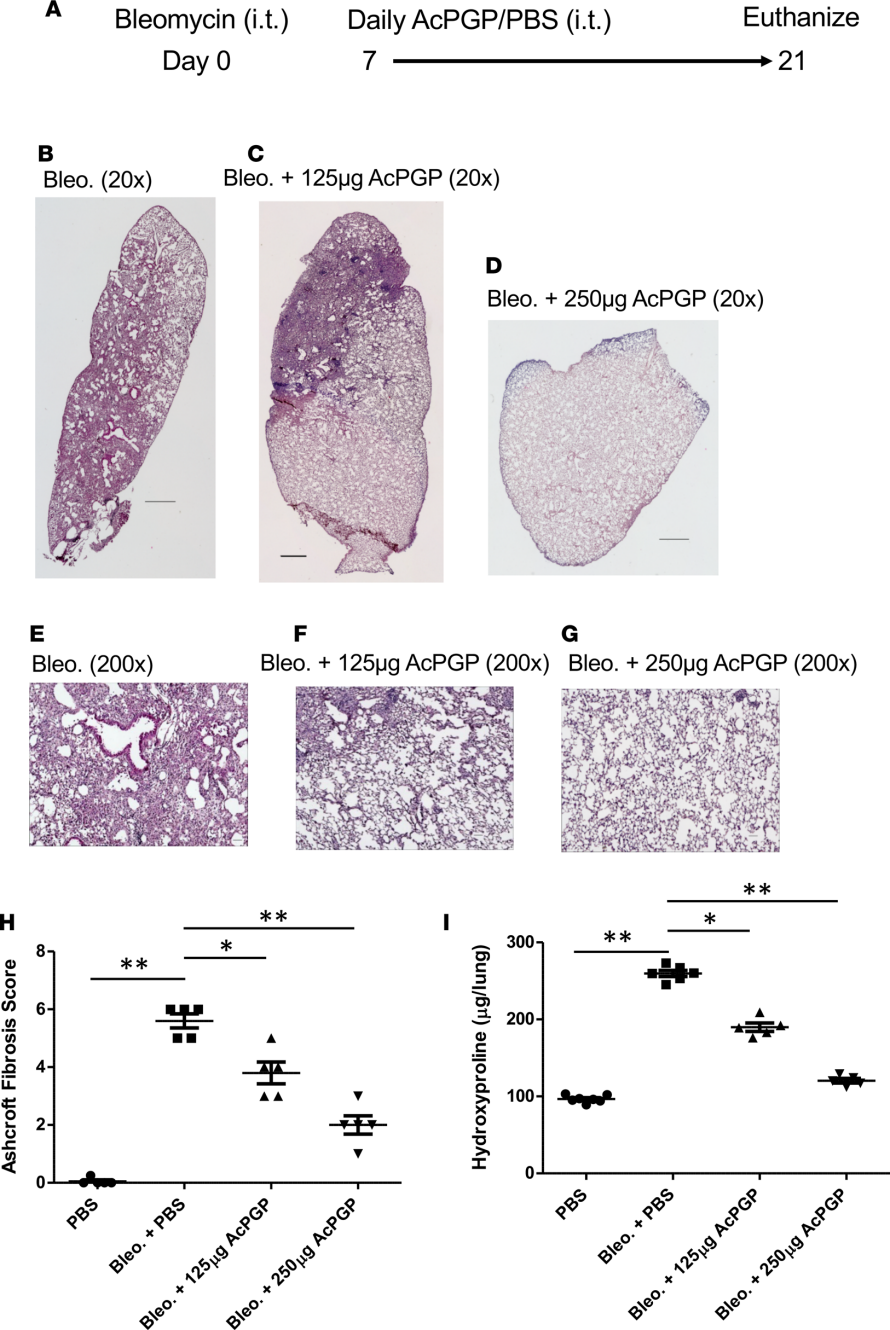
AcPGP inhibits bleomycin-induced pulmonary fibrosis. C57BL/6 mice were administered bleomycin i.t. followed after a week by daily i.t. administrations of 125 μg or 250 μg AcPGP per mouse or PBS for 2 weeks (**A**). Representative H&E stained lung sections (**B–G**) and quantification of the fibrosis using the Ashcroft scoring system (**H**). Images are 20× and 200×, as indicated; scale bars: 500 μM. (**I**) Total lung hydroxyproline measurements. Experiments are from at least 5 mice/group and representative of 2 independent experiments. Results depicted as mean ± SEM. *P < 0.05 or **P < 0.01 using Kruskal-Wallis with Dunn’s post test.

**Figure 7:**
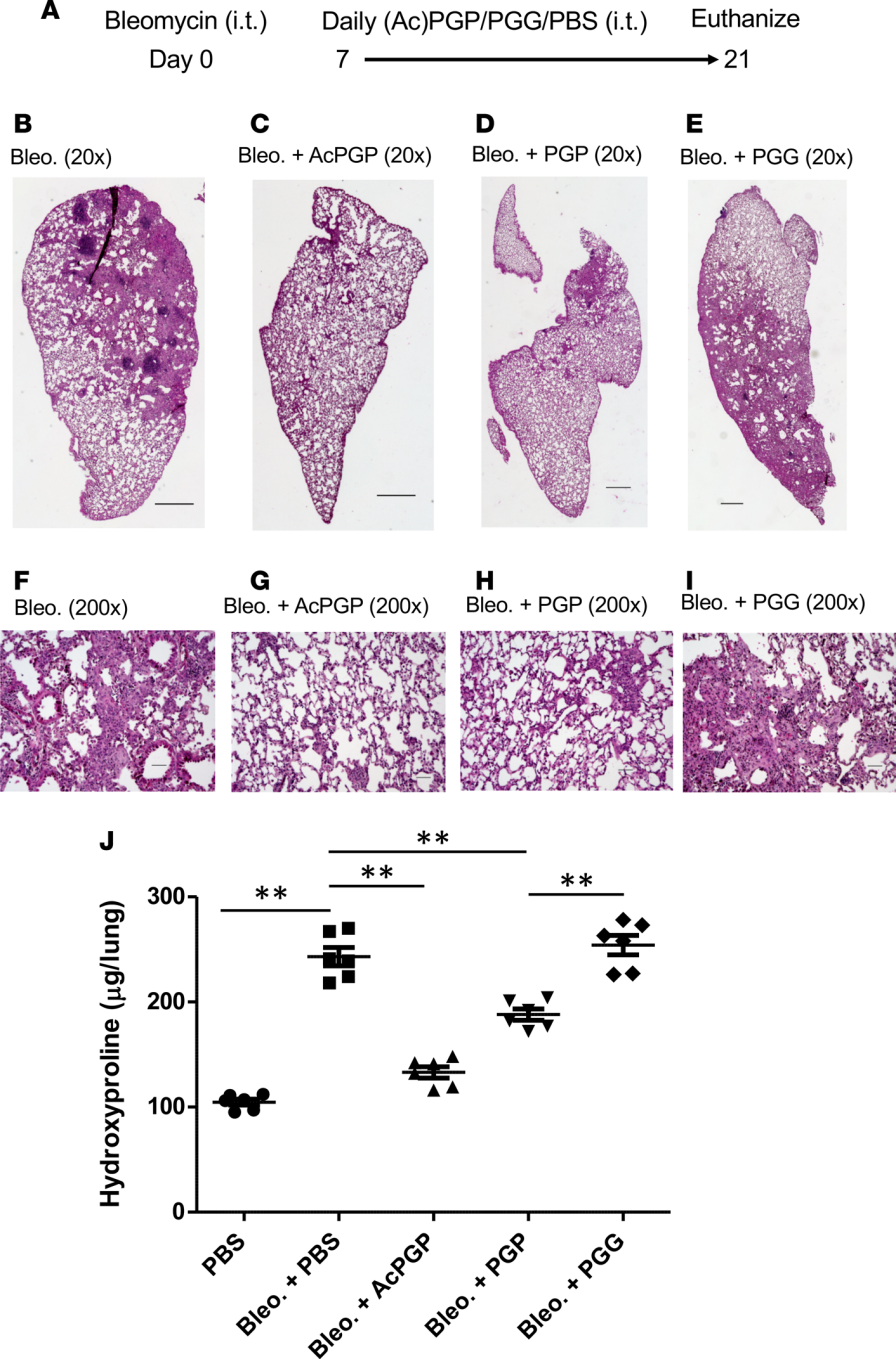
Efficacy of peptide analogs of AcPGP against fibrosis. C57BL/6 mice were administered bleomycin i.t. followed after a week by daily i.t. administrations of 250 μg AcPGP, PGP, PGG, or PBS for 2 weeks (**A**). Representative H&E-stained lung sections (**B–I**). Images are 20× and 200×; scale bars: 500 μM. (**J**) Total lung hydroxyproline measurements. Experiments are from at least 5 mice/group and representative of 2 independent experiments. Results depicted as mean ± SEM. **P < 0.01 using Kruskal-Wallis with Dunn’s post test.

Given the pathological role of ACE ascribed to the development of IPF, we questioned whether the robust AcPGP degradation observed could paradoxically be pathological in the setting of IPF. To test this hypothesis, AcPGP was administered i.t. daily for 14 days (starting at day 7 after bleomycin to coincide with the onset of repair) to the lungs of bleomycin-exposed mice at 2 different doses (250 μg or 125 μg), with mice being euthanized at day 21 (Figure 6A). Strikingly, the administration of 250 μg AcPGP per day almost completely prevented bleomycin-induced pulmonary fibrosis, with 125 μg per day also proving significantly effective (Figure 6, B–H). These histological findings of reduced fibrosis were confirmed by whole lung hydroxyproline measurements, which were also strikingly reduced in bleomycin-treated animals (Figure 6I). As a point of note and in keeping with an improved health status, bleomycin-exposed, AcPGP-treated mice also failed to lose weight, while PBS-treated mice lost weight as expected (24% ± 5% for PBS vs. 1.3% ± 1.2% for AcPGP; mean ± SEM). To assess the relative potencies of AcPGP and PGP, bleomycin-exposed mice were subsequently treated with 250 μg AcPGP, PGP, or control peptide PGG for 2 weeks starting at day 7 (Figure 7A). Administration of AcPGP again largely prevented the development of pulmonary fibrosis, with PGP being slightly less effective and PGG being completely ineffective (Figure 7, B–I). Again, histological findings were reflected by whole-lung hydroxyproline measurements (Figure 7J). These data indicate that the antifibrotic effect is mediated by the PGP tripeptide with N-terminal acetylation conferring greater potency, as previously reported with neutrophil chemotaxis (19). PGP may also be less effective in vivo, as it is rapidly degraded in the lung by LTA_4_H, whereas AcPGP is not (17).

**Figure 8:**
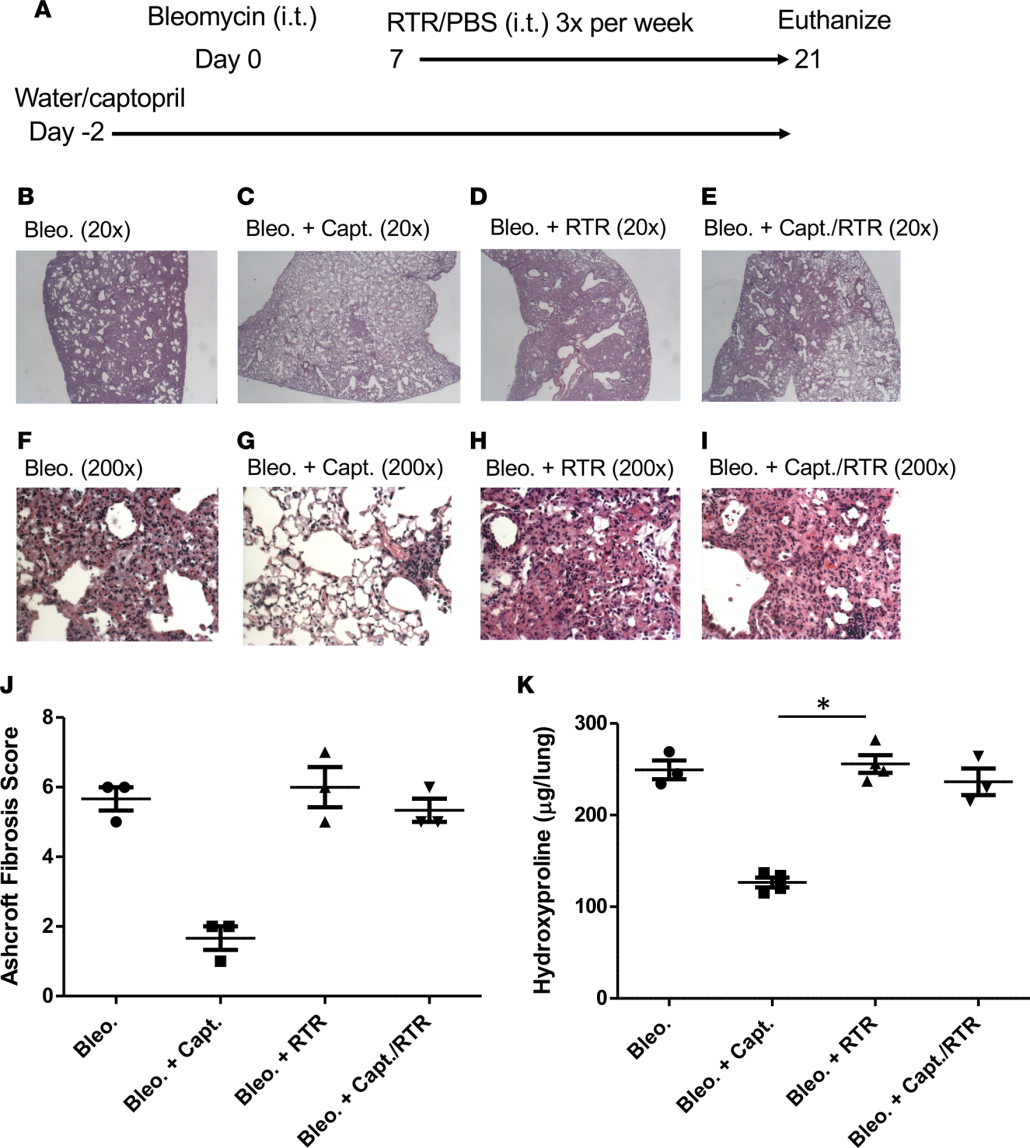
A PGP antagonist abrogates the antifibrotic effects of captopril. C57BL/6 mice were provided with water or water supplemented with captopril and administered bleomycin i.t., followed a week later by i.t. administrations of 50 μg RTR or PBS 3 times a week for 2 weeks (**A**). Representative H&E-stained lung sections (**B–I**) and quantification of the fibrosis using the Ashcroft scoring system (**J**). Images are 20× and 200×. (**K**) Total lung hydroxyproline measurements. Experiments are from 3–5 mice/group and representative of 2 independent experiments. Results depicted as mean ± SEM. *P < 0.05 using Kruskal-Wallis with Dunn’s post test.

Finally, we looked to directly ascertain whether the pathological role attributed to ACE in the development of fibrosis was specifically a consequence of its ability to efficiently degrade AcPGP. Therefore, bleomycin-treated mice were administered the ACE inhibitor captopril in their drinking water (starting 2 days prior to bleomycin exposure) with or without AcPGP antagonist arginine-threonine-arginine (RTR) (47) (Figure 8A). Bleomycin-treated mice administered captopril were largely protected from development of fibrosis (Figure 8, B–J), with a significant reduction in lung hydroxyproline content (Figure 8K). However, the protective effect of captopril was lost when mice were concomitantly administered RTR (Figure 8), clearly demonstrating that protection afforded by ACE inhibition is attributable to AcPGP accumulation.

## Discussion

We have previously demonstrated that the enzyme LTA_4_H is able to potently degrade the neutrophil chemoattractant PGP to ensure the efficient resolution of inflammation (17) and that this system is perturbed in chronic lung diseases, enabling PGP accumulation and persistent neutrophilia (17, 21). However, the acetylated variant AcPGP is completely protected from LTA_4_H-mediated degradation (17). Accordingly, it is clear that the acetylation process is a critical event in defining bioavailability and pathogenesis of this matrikine, with an abundance of AcPGP observed in COPD and CF (14, 15, 21–24). In this study, we identify a potentially novel pathway by which AcPGP can be degraded through an action of the enzyme ACE. We demonstrate that pulmonary ACE is elevated during episodes of acute inflammation, likely as a consequence of enhanced vascular permeability, to ensure the efficient degradation of AcPGP. Conversely, we anticipate that this pathway may be aberrant in COPD, enabling the accumulation of AcPGP in this disease. Surprisingly, we subsequently identify a protective role for AcPGP in limiting pulmonary fibrosis, and we demonstrate the pathogenic function attributed to ACE in fibrosis to be a consequence of overzealous AcPGP degradation. Thus, AcPGP can seemingly have very divergent roles: pathogenic in its capacity to drive neutrophilic inflammation and ECM degradation in the context of COPD, but protective in its capacity to limit fibrotic lung disease observed in IPF. Thus, we hypothesize that while COPD and IPF may be diseases with similar etiologies, they ultimately manifest as very different pathologies as a consequence of disparate perturbations of the AcPGP-ACE axis.

The renin-angiotensin system (RAS) controls blood pressure and renal homeostasis (48), with the enzyme ACE functioning as the distal enzyme in a cascade that generates AngII from AngI (49). AngII is a powerful vasoconstrictor and, thus, is critical in the regulation of blood pressure (50). While ACE classically functions to generate AngII, it is acknowledged to be a promiscuous enzyme with the capacity to act on a variety of natural substrates, and it possesses physiological roles beyond its capacity to modulate blood pressure (51). Of note, it has been demonstrated that ACE has the capacity to degrade AcSDKP, a tetrapeptide that has been shown to be capable of suppressing fibrosis (52–55). We now identify AcPGP as a peptide substrate for ACE and highlight a capacity for the AcPGP-ACE axis in differentially regulating inflammation and fibrosis.

ACE is tethered to the luminal side of the endothelium, with circulating plasma ACE derived from proteolytic cleavage (56), rationalizing the high levels of AcPGP-degrading activity and ACE seen in our studies in the blood and highly vascularized organs such as the lungs. To degrade ECM collagen–derived AcPGP within the lung, ACE must be freely available in an extracellular environment. We have presented evidence suggesting that BALF ACE is elevated during episodes of inflammation as a consequence of increased vascular permeability and an influx of plasma enzyme. Given that ACE is expressed by multiple cell types that are prevalent within the fibrotic lung, we cannot, however, disregard the possibility that there is also a localized release of ACE from resident and infiltrating cells. ACE exists as a single polypeptide chain, with 2 homologous but independent N- and C-terminal catalytic domains (57). Both catalytic sites of ACE can hydrolyze AngI, but only the N-domain site can degrade atypical substrates such as AcSDKP (53, 58–60). Captopril has a higher affinity for the N-domain site of ACE (61) and accordingly inhibits hydrolysis of AcSDKP with lower inhibitory constant (*K_i_*) than AngI (62). Since captopril also inhibited hydrolysis of AcPGP more than an AngI-like substrate, AcPGP cleavage is also likely to be restricted to the N-domain.

Striking similarities exist between the LTA_4_H-PGP axis we have previously described (17) and the ACE-AcPGP axis described in this study. Both LTA_4_H and ACE are dual-functioning enzymes with complex roles in health and disease. LTA_4_H classically functions to generate the proinflammatory lipid mediator LTB_4_, a function seemingly at odds with its antiinflammatory PGP-degrading activity (17). ACE is a truly promiscuous enzyme that has multiple peptide substrates with pleiotropic physiological functions (51), though degradation of AcPGP is the first truly antiinflammatory activity ascribed to this enzyme. In this study, we demonstrate that PGP is normally degraded by LTA_4_H and that PGP accumulation (and hence failure of the LTA_4_H system) is a requisite for conversion to AcPGP, with ACE subsequently functioning to degrade any of the acetylated variant that is generated. Thus, it would appear that 2 enzymes act in tandem during episodes of acute inflammation to ensure that no PGP or AcPGP are allowed to persist and that neutrophilia is resolved. AcPGP has recently been demonstrated to function to promote vascular permeability via direct interaction with endothelial cells (63), and thus, the peptide can itself indirectly promote its own degradation through increasing availability of tissue ACE. Given the role ascribed to AcPGP in this study in limiting fibrosis, it should be considered that fibrosis is actually a repair process gone wrong. Under physiological conditions, it may be that AcPGP functions to limit any repair during early/ongoing inflammation, but as AcPGP is degraded by ACE and inflammation resolved, a repair process is initiated.

Levels of PGP/AcPGP are elevated in murine cigarette-smoke models of emphysema (21, 64) and clinical samples from smokers and COPD patients (21–24), wherein the accumulation of peptide can drive neutrophil persistence and protease imbalance. Given the role ascribed to AcPGP/PGP in this study in limiting fibrosis, it is plausible that accumulations of these peptides in COPD patients may not only perpetuate inflammation, but also prevent reparative processes from taking place. We have previously demonstrated that reactive aldehydes within cigarette smoke are able to inhibit the PGP-degrading activity of LTA_4_H, rationalizing the accumulation of PGP in COPD patients and murine models (17, 21). Furthermore, the same reactive aldehydes are also capable of acetylating PGP (to AcPGP) and, thus, protecting it from LTA_4_H-mediated degradation (18). It is feasible that the ACE pathway is also aberrant in COPD, and from our cohort of patients, there is the suggestion that BALF ACE levels are reduced in COPD patients relative to healthy controls and IPF. Previous studies into the effect of cigarette smoke on ACE levels/activity have been inconsistent and conflicting, though a reduction in ACE levels (65) and activity (66) have been reported in smokers relative to nonsmokers, with activity increased again by smoking cessation.

Increasingly, it is apparent that elevated ACE levels are associated with aberrant repair and fibrosis, though the mechanisms defining the pathogenesis are poorly defined. Increased ACE expression in pulmonary granulomas in sarcoidosis has long been recognized (67), and elevations in ACE have been reported in IPF (40–42). The classical product of ACE, AngII, has been suggested to play a role in tissue fibrosis through upregulation of collagen gene expression in lung fibroblasts and apoptosis of alveolar epithelial cells (40, 68). Accordingly, some studies have reported a partial amelioration of bleomycin-induced pulmonary fibrosis following administration of angiotensin II receptor type 1 receptor antagonists such as losartan (69–71). However, other studies have failed to confirm these protective effects of AngII receptor antagonism (72), and it is increasingly anticipated that other activities of ACE may be integral in defining fibrosis. AcSDKP has been suggested to be antifibrotic, and thus, the pathological role ascribed to ACE could be attributable to its capacity to degrade this peptide. Mice deficient in the N-domain of ACE were protected from bleomycin-induced lung fibrosis. Though AcSDKP could not be detected in the lungs of the mice, the protective effect was ascribed to preservation of this peptide (43). To support this notion, inhibition of the enzyme PE that is critical to the generation of AcSDKP restored the fibrotic phenotype (73). However, PE is also critical for PGP generation, and hence, the aforementioned protective phenotype of ACE N-domain–KO mice could be attributed to a failure of ACE to degrade AcPGP. Accordingly, we have demonstrated that administration of AcPGP to the lungs of mice dramatically and dose-dependently reduced collagen accumulation and fibrosis. Furthermore, we demonstrated that the capacity of the ACE inhibitor, captopril, to ameliorate bleomycin-induced pulmonary fibrosis was entirely dependent on AcPGP accumulation, since protection was abolished by the AcPGP antagonist RTR. The mechanism whereby AcPGP prevents fibrosis in vivo is yet to be defined but could be due to proteolytic degradation of the ECM, given the capacity of PGP accumulation to drive protease imbalance in the lung (25). Alternatively, AcPGP may possess unappreciated roles in defining wound repair and fibrosis by modulating processes such epithelial cell apoptosis, fibroblast recruitment/differentiation, collagen synthesis/deposition, or collagen removal by macrophages. Elucidating this pathway would provide additional ways of influencing the ACE-AcPGP axis and suggest therapeutic approaches for IPF.

While previous studies have largely ascribed a predominantly pathological role to PGP/AcPGP in diseases such as COPD and CF, it now seems likely that this peptide in moderation serves a beneficial role in limiting excessive collagen deposition. COPD and IPF are thought to arise from common etiologies and yet manifest with quite disparate disease pathologies. Is it feasible that divergences in the ACE-AcPGP axis define the relative progression of disease? If PGP/AcPGP is allowed to persist, as in COPD, then it can drive a vicious cycle of neutrophilic inflammation and protease imbalance, while simultaneously preventing repair. Conversely, if PGP/AcPGP are degraded too efficiently, as may be the case in IPF, then no signal is present to limit collagen deposition; hence, fibrosis ensues. Levels of PGP/AcPGP generation and degradation will ultimately be key in defining this balance, which in turn are seemingly dictated by genetic and environmental influences. In this manner, differences in ACE expression and activity between individuals could explain differing susceptibilities to smoking-induced lung diseases and phenotypes via modulating levels of AcPGP.

## Methods

### Mice.

Six- to 8-week-old female BALB/c mice were purchased from Harlan Laboratories. Six- to 8-week-old female C57BL/6 mice were purchased from The Jackson Laboratory. Lta4h^–/–^ mice and littermate controls were on a 129/S6 background and bred in house at Imperial College London, with female 6- to 8-week-old mice utilized in experiments. ACE 1 mice (null for ACE) (74) and ACE 14/14 mice (null for somatic ACE) (provided by Ken Bernstein, Cedars-Sinai Medical Center, Los Angeles, CA, USA) and respective littermate controls were bred in house at Sinai-Cedars Medical Centre, with plasma from 11- to 20-week-old female and male mice utilized in experiments. All mice were kept in specified pathogen-free conditions and provided autoclaved food, water, and bedding.

Lta4h^–/–^ mice were genotyped by PCR on genomic DNA extracted using the Extract-N-Amp Tissue PCR Kit (MilliporeSigma). The WT expression of LTA_4_H was detected by primers oIMR1720 (5′ - CGA ATC CAT GCT TAA AAT TGC - 3′) and oIMR1721 (5′ - GCG TTA CGA ACG TGA GAC AA - 3′) to yield a product size of 128 bp, and Lta4h^–/–^ LTA_4_H was detected by primers oIMR6916 (5′ - CTT GGG TGG AGA GGC TAT TC - 3′) and oIMR6917 (5′ - AGG TGA GAT GAC AGG AGA TC - 3′) to yield a product size of 280 bp. Amplification was achieved by PCR (94°C for 3 min > 35 × 2.5 min cycles [30 sec at 94°C > 60 sec at 60°C > 60 sec at 72°C] > 72°C for 2 min). Amplification fragments were visualized on agarose gels (2%).

### Mouse challenge models.

Balb/c mice were anesthetized with isoflourane and treated i.n. with 50 μg/kg recombinant murine MIP-2 (Peprotech) or 500 μg/kg LPS (ultrapure from *E*. *coli* K12 strain; InvivoGen), both in 50 μl PBS, and culled at times indicated in the text. In some experiments, Balb/c mice received 250 mg/kg histamine (MilliporeSigma) i.v. in 200 μl PBS and were culled 30 minutes later. In other experiments, Lta4h^–/–^ mice and littermate controls were anesthetized with isoflourane and treated i.n. with 500 μg/kg LPS in 50 μl PBS. These mice were also administered i.p. captopril (100 mg/kg; MilliporeSigma) or 200 μl PBS (control) at the time of LPS administration and 24 hours prior.

Bleomycin sulphate (MilliporeSigma) dissolved in 50 μl sterile saline was administered i.t. to C57BL/6 mice at a dose of 1 U/kg. Control mice received saline. In some experiments, mice were also administered 250 μg or 125 μg AcPGP/PGP (Bachem) dissolved in 50 μl PBS or PBS i.t. every day for 2 weeks starting a week after bleomycin exposure. In other experiments, mice were provided with drinking water supplemented with captopril (500 mg/l; MilliporeSigma) starting 2 days prior to administration of bleomycin, and were subsequently administered 50 μg RTR (Bachem) dissolved in 50 μl PBS or PBS i.t. 3 times per week for 2 weeks starting a week after bleomycin exposure.

### Cell recovery and isolation.

Mice were administered 150 mg/kg pentobarbital and exsanguinated via the femoral artery. Serum was isolated by centrifugation for 8 minutes at 5,000 *g* and frozen at –80°C until required. To obtain plasma, blood was collected via cardiac puncture and diluted 10-fold into Buffered Citrate (60 mM sodium citrate/40 mM citric acid), and then centrifuged 2 times at 800 *g* for 10 minutes with supernatant collected. To perform broncholaveolar lavage, the lungs were then inflated 5 times with 1.5 ml PBS via an i.t. cannula, and the resulting BALF was centrifuged once at 400 *g* and the supernatant stored at –80°C. Right superior lung lobes, spleen, liver lobe, and a segment of the small intestine dissected from naive Balb/c mice were snap frozen at the time of harvest. These tissues were subsequently homogenized in PBS at a concentration of 50 mg/ml. Homogenates were centrifuged at 800 *g* for 10 minutes, and supernatant was collected for downstream analysis.

### Human BALF.

BALF from human subjects with COPD were remnants obtained from a previous clinical trial (75). Subjects (Table 1) aged 40–80 years, smokers and nonsmokers, were categorized as COPD or controls based on the presence or absence of airflow obstruction (FEV_1_/FVC below the lower limit of normal for age, race, sex, and height). A minimum of 10 pack years of tobacco use was required for patients with COPD. COPD patients were on inhaled bronchodilators and/or corticosteroids only. Exclusion criteria were asthma, bronchiectasis, or other lung disease and any change in respiratory medications or respiratory illness in the month prior to enrollment. BALF from subjects with IPF were remnants from specimens obtained for clinical purposes. IPF (Table 1) was defined as an appropriate clinical presentation with usual interstitial pneumonia (UIP) seen on high-resolution chest CT scans that were absent other causes of interstitial lung disease (76). In some cases, UIP was confirmed by surgical lung biopsy. Subjects with IPF had stable disease and were not taking oral corticosteroids, immune suppressants, perfenidone, or nintedanib.

### Assessment of fibrosis in bleomycin-exposed mice.

At the end of the treatment period, mice were euthanized and lungs excised and inflated with 4% paraformaldehyde in PBS for histochemical analysis by H&E. The Ashcroft scoring system was used to assess the pulmonary fibrotic changes in lung tissue sections (77). Two independent and blinded observers determined the Ashcroft fibrosis score for each experimental group from at least 3 individual experimental mice of each group. Whole-lung collagen levels were determined by assessment of lung hydroxyproline. The harvested lungs were hydrolyzed in 6 mol/l HCl at 110°C for 24 hours, and the amount of hydroxyproline in the lung acid–hydrolysates was determined by colorimetric assay, as previously described (78).

### AcPGP degradation experiments.

BALF (neat), serum (diluted 1/10 in PBS), tissue homogenate (diluted 1/10 in PBS), or recombinant porcine ACE and prolidase (at concentrations defined in the text; MilliporeSigma) were incubated with 1 μg/ml (BALF) or 100 μg/ml (all other tissues and recombinant proteins) AcPGP at 37°C 5% CO_2_ for varying periods of time (as defined in the Figure 1–3 and 5 legends). Concentrations of AcPGP remaining were subsequently quantified by liquid chromatography–electrospray ionization-tandem mass spectrometry (ESI-LC/MS/MS; as discussed below) by comparison with AcPGP standards. The percentage of peptide degraded was determined relative to control samples of AcPGP alone. In some instances, AcPGP degradation was expressed as μg peptide degraded/mg of total tissue protein or per ml of biological fluid. AcPGP degradation was also assessed by measurement of free proline released and its ensuing reaction with ninhydrin (as discussed below). In some experiments, serum was preincubated with EDTA, AEBSF, PMSF, E64, antipain (all 1 μM; MilliporeSigma), captopril, or enalopril (as concentrations defined in the text; MilliporeSigma) for 30 minutes at 3°C 5% CO_2_ prior to addition of AcPGP.

### ESI-LC/MS/MS for PGP detection.

For peptide quantification in BALF, PGP, and AcPGP were measured using a MDS Sciex API-4000 spectrometer (Applied Biosystems) equipped with a Shimadzu HPLC. For peptide quantification from degradation experiments, AcPGP was measured using a Thermo Accela Pump and Autosampler coupled to a Thermo TSQ Quantum Access. HPLC was done using a 2.0 × 150 mm Jupiter 4 μm Proteo column (Phenomenex) with (buffer A) 0.1% HCOOH and (buffer B) MeCN + 0.1% HCOOH for 0–0.5 min 5% buffer B/95% buffer A, then increased over 0.5–2.5 min to 100% buffer B/0% buffer A. Background was removed by flushing with 100% isopropanol/0.1% formic acid. Positive electrospray mass transitions were at 270–70, 270–116, and 270–173 for PGP and 312–140 and 312–112 of AcPGP. Peak area was measured, and PGP/AcPGP peptide concentrations were calculated using a relative standard curve method, as previously described (12).

### Measurement of free proline.

Aliquots from PGP degradation experiments were diluted 1:10 in PBS (to a final volume of 250 μl). Glacial acetic acid (250 μl) was then added, followed by 250 μl of ninhydrin solution (25 mg/ml in acetic acid/6 M phosphoric acid; heated at 70°C to dissolve). The reaction mixture was heated at 100°C for 60 minutes and allowed to cool to room temperature, the proline containing fraction was extracted with 500 μl of toluene, and optical density was measured at 520 nm.

### ACE quantification.

The levels of ACE in BALF and tissues was measured by ELISA per the manufacturer’s directions (Duo Set; R&D Systems).

### Albumin quantification.

Suitably diluted mouse BALF was assessed for albumin (Bethyl Laboratories) by ELISA, following the manufacturer’s instructions.

### Statistics.

Mouse data were assessed for normal distribution using the Shapiro-Wilk normality test and found to be nonnormal. For comparison between 2 groups, statistical significance was calculated with a nonparametric Mann-Whitney test (2-sided). Group comparisons were performed using a nonparametric ANOVA test (Kruskal-Wallis), followed by a Dunn’s post test for multiple comparisons between groups. Details of specific statistical test used for each experiment are described in respective figure legends. Results are depicted as mean ± SEM unless stated otherwise. Correlations were calculated using a Spearman’s Rank Correlation coefficient for nonparametric data. Assessment of statistical significance of ACE levels in human BALF was performed by a parametric unpaired t test. All *P* values of ≤ 0.05 (*) and ≤ 0.01 (**) were considered significant and are referred to as such in the legends. Graph generation and statistical analyses were performed using GraphPad Prism software (GraphPad Software Inc.).

### Study approval.

For animal experiments conducted at Imperial College London, research was carried out in accordance with the recommendations in the Guide for the Use of Laboratory Animals of Imperial College London. All animal procedures and care conformed strictly to the UK Home Office Guidelines under the Animals (Scientific Procedures) Act 1986, and the protocols were approved by the Home Office of Great Britain. Animal experiments at the University of Alabama at Birmingham were performed in accordance with the NIH guidelines for the IACUC. Plasma samples obtained from WT and ACE-KO animals followed protocols approved by the Cedars-Sinai IACUC. Human BALF samples were from a repository approved by the IRB of the University of Alabama at Birmingham. All subjects provided written, informed consent.

## Author contributions

PJO, QD, RJS, and JEB designed and interpreted the experiments and prepared the manuscript. RJS, with SA and DFP, performed experiments detailing the activity of ACE and its role in acute pulmonary inflammation. PJO and QD conducted experiments in bleomycin-exposed mice, with the assistance of GC, LV, KRG, MLL, and MR. KEB and EAB provided plasma from ACE-KO mice and respective controls. PLJ aided with mass spectrometry, and PLJ and CML contributed discussions throughout the work.

## Figures and Tables

**Table 1:**
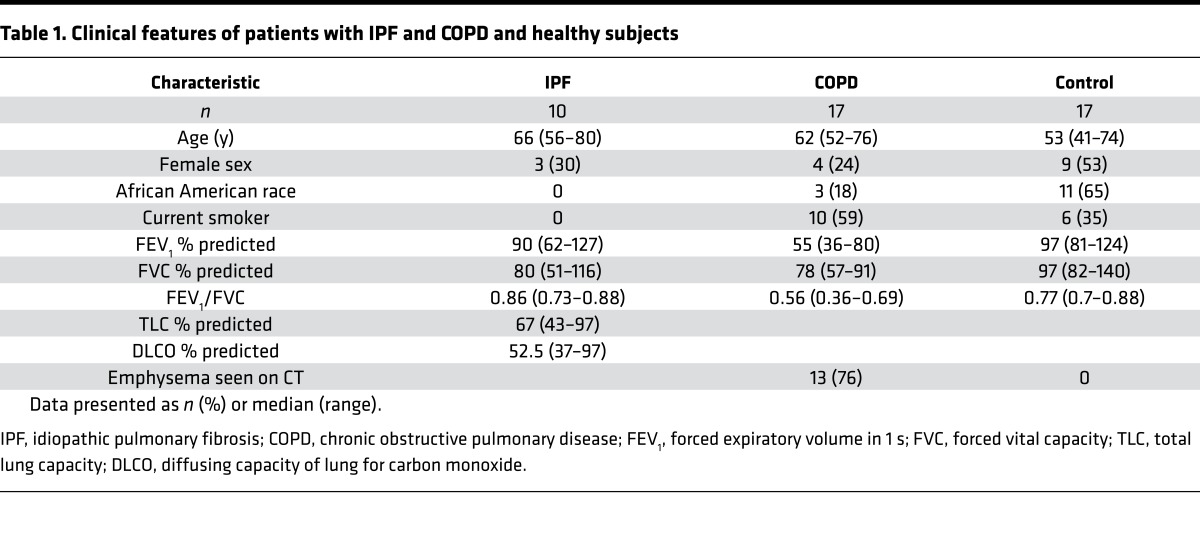
Clinical features of patients with IPF and COPD and healthy subjects

## Version 1 11/16/2017

Electronic publication
